# Explicit and Implicit Affect and Judgment in Schizotypy

**DOI:** 10.3389/fpsyg.2019.01491

**Published:** 2019-07-01

**Authors:** Elizabeth A. Martin, Jessica P. Y. Hua, Kelsey T. Straub, John G. Kerns

**Affiliations:** ^1^Department of Psychological Science, University of California, Irvine, Irvine, CA, United States; ^2^Department of Psychological Sciences, University of Missouri, Columbia, MO, United States

**Keywords:** positive emotion, negative emotion, IPANAT, social anhedonia, perceptual aberrations, magical ideation

## Abstract

Although emotion deficits in schizotypy have been reported, the exact nature of these deficits is now well understood. Specifically, for social anhedonia (SocAnh), there are questions about whether any decrease in positive affect only reflects an explicit bias not observed in other measures (e.g., implicit affect measure). At the same time, for individuals with elevated levels of perceptual aberrations or magical ideation (PerMag), there is some evidence of an increased influence of affect on judgment. It is also possible that the influence of implicit affect on judgment might be especially pronounced in PerMag; however, this has not been previously examined. The current study involved people with elevated levels of SocAnh (*n* = 95), elevated levels of PerMag (*n* = 62), and people with average or lower levels of both (*n* = 246). We found that SocAnh was associated with decreases in both explicit and implicit positive affect. We also found that PerMag was related to stronger relationships between implicit affect, both positive and negative, and a judgment task. These results suggest that decreased positive affect is a core feature of SocAnh and that a heightened influence of affect could be related to the development of peculiar beliefs/experiences associated with PerMag.

## Introduction

Schizotypy is a personality organization characterized by the presence of schizophrenia-like symptoms reflecting vulnerability to develop schizophrenia (Meehl, 1962, 2001). There is evidence that schizotypy is associated with emotion deficits (Horan et al., 2008; Martin et al., 2011). At the same time, there is evidence that schizotypy is multidimensional and includes a negative and positive dimension (Kwapil et al., 2008). Furthermore, emotion is multifaceted (Barrett et al., 2007), with distinctions between positive vs. negative emotions (Watson et al., 1988) and between implicit and explicit emotional experiences (Quirin et al., 2009a). Given their direct influence on social and functional outcomes (e.g., Blanchard et al., 1998), the current study sought to further clarify the nature of these emotion facets among individuals reporting elevated levels of negative schizotypy [characterized by extremely elevated levels of social anhedonia (SocAnh)] or positive schizotypy (characterized by extremely elevated frequency of perceptual aberrations or magical ideation; PerMag) in order to inform potential prevention and intervention strategies.

Psychopathologists have suggested that emotion traits might be important for schizotypy. SocAnh is characterized by diminished self-reported experience of positive emotion (e.g., Brown et al., 2007; Kerns et al., 2008; Martin et al., 2011) as well as increased self-reported experience of negative emotion (e.g., Gooding et al., 2002; Gooding and Tallent, 2003; Blanchard et al., 2011). SocAnh is associated with an increased risk of schizophrenia-spectrum personality disorders (Kwapil, 1998; Gooding et al., 2005), and for individuals with a spectrum disorder, it is poorly treated (Horan et al., 2006). Thus, understanding the nature of emotional abnormalities in SocAnh could potentially help prevent the onset of a schizophrenia-spectrum disorder as well as help treat functional disability in those with a spectrum disorder. However, the nature of deficits in these emotion mechanisms in at-risk populations is still unclear (e.g., Kring and Moran, 2008).

One area that lacks clarity relates to evidence of decreased trait positive affect in SocAnh (e.g., Horan et al., 2008). Specifically, there are questions about how to interpret this decrease in positive affect because of an apparent “objective–subjective deficit paradox” in groups characterized by elevated SocAnh. That is, on subjective measures, these groups often report experiencing deficits while smaller deficits are sometimes found on objective measures of the same constructs [Chun et al., 2013; Cohen et al., 2014; Mitchell and Cohen, 2017; Li et al., 2019; cf. Tallent and Gooding (1999), Gooding et al. (2006), and Ettinger et al. (2015) for a review]. It has been argued that this also calls into question the nature of any decreased positive affect on explicit measures (e.g., self-report assessments that directly ask participants questions regarding their subjective emotional experiences; Cohen et al., 2017). Given this, it is possible that decreased positive affect in SocAnh might only be found in explicit ratings of positive affect. However, positive affect can also be assessed with implicit measures of affect (i.e., measures that indirectly assess one’s emotional experience).

Part of the rationale for assessing affect implicitly is that it is often thought that emotion reflects loosely coupled changes in feelings, thoughts, physiology, and behavior (e.g., Barrett, 2006). Hence, an implication of this loose coupling is that there is low convergence between explicitly reported affect and other aspects of emotion (e.g., Mauss et al., 2005; Lindquist and Barrett, 2012). Therefore, implicitly measured affect could reflect aspects of emotion that are not captured by explicit affect ratings. Consistent with this, implicit measures of affect have been found to predict outcomes over and above explicit affect measures. For example, while explicit affect ratings generally show at best low convergence with physiological responses (e.g., Mauss and Robinson, 2009; Quigley and Barrett, 2014), responses on the Implicit Positive and Negative Affect Test (IPANAT) (Quirin et al., 2009a) have been reported to be better predictors of physiological responses to a stressor (e.g., cortisol release; Quirin et al., 2009b). Thus, in the current research, we examined whether SocAnh would be associated with a decrease in both current explicit and implicit positive affect or instead by a decrease only in current explicit positive affect, a previously unexplored, possible distinction.

Similar to the link between SocAnh and emotional disturbances, it has been suggested that emotion disturbances might foster the development of peculiar beliefs and experiences (Berenbaum et al., 2003, 2006). Given that individuals with elevated PerMag are at an increased risk for psychosis (Lenzenweger, 1999), understanding which emotion facets are associated with beliefs and experiences might suggest how emotion could contribute to psychosis and psychotic-like symptoms. In previous research, PerMag has been associated with increased self-reported influence of emotion (Cicero and Kerns, 2010). Also consistent with this, there is psychophysiological data of increased reactivity to both positive and negative affect in PerMag (Karcher and Shean, 2012; Martin et al., 2017). Hence, this suggests that abnormalities in PerMag might reflect an increased influence of affect.

Despite this evidence, it is unclear whether and how affect does influence judgment in PerMag. It is possible that implicit affect might be especially likely to influence judgment in PerMag. According to the theoretical model outlined by Risen (2016), odd beliefs and experiences could reflect an increased influence of System 1 thinking, which is thinking that reflects use of heuristics and automatic spreading of activation and that is therefore susceptible to many cognitive biases. Further, an inability or unwillingness to attempt to override System 1 thinking by the use of more effortful and rational System 2 thinking could also foster odd beliefs and experiences (Risen, 2016). Importantly, according to Quirin and Bode (2014), implicit affect is thought to arise due to automatic activation of affective representations within System 1 thinking. Hence, implicit affect should increase the influence of System 1, which according to Risen (2016) should foster the development of PerMag. However, previous research has yet to examine whether PerMag is associated with an increased influence of implicit affect on judgment. Thus, the current study examined whether PerMag might be associated with an increased influence of affect on judgment.

In the current study, a group with extreme levels of SocAnh, a group with extreme levels of PerMag, and a control group completed explicit and implicit affect measures as well as a judgment task. Overall, compared to a control group, we expected that SocAnh and PerMag groups would show differential emotion-related abnormalities. Specifically, if SocAnh is associated with decreased positive affect, we would predict the SocAnh group would report both decreased explicit and implicit positive affect compared to the other groups. In contrast to SocAnh, if PerMag is associated with an increased influence of affect, we would expect the PerMag group would exhibit an increased influence of both positive and negative implicit affect on judgment compared to the other groups.

## Materials and Methods

### Participants

We used an extreme-groups approach (Preacher et al., 2005) that compared people with elevated SocAnh and people with elevated levels of PerMag to a control group. Participants were undergraduates from a large university who participated for Introduction to Psychology course credit. Participants could sign up for study time slots on a scheduling website. In order to ensure a large enough group with elevated SocAnh or PerMag, we also recruited from students who completed an online departmental mass testing (*N* = 2,025; participants completed the mass testing as part of course credit and knowing it would make them eligible for studies), with mass testing including a subset of SocAnh – 15 items from the Revised SocAnh Scale (Eckblad et al., 1982) – and PerMag items – 8 items each from the Perceptual Aberration Scale (Chapman et al., 1978) and from the Magical Ideation Scale (Eckblad and Chapman, 1983). In the laboratory, participants completed the full version of these scales, and inclusion in the current study was based on their scores on the full version using cut-offs scores obtained from a previous large-sample study (Kerns and Berenbaum, 2000). In addition, participants completed the Chapman Infrequency Scale (Chapman and Chapman, 1983) to screen for careless or invalid responses. Based on previous research (e.g., Chapman et al., 1994), those who endorsed 3 or more items on this 13-item, true–false scale was eliminated from analyses.

There were 95 people in the SocAnh group (68.4% women, mean age 19.2 years, *SD* = 2.85, 71.6% Caucasian) who scored above 1.96 *SD*s above the same-sex mean on the Revised SocAnh Scale. There were 62 people in the PerMag group (51.6% women, mean age 18.94 years, *SD* = 0.99, 75.8% Caucasian) who scored above 1.96 *SD*s above the same-sex mean on the Perceptual Aberration or Magical Ideation scales or had a summed, standardized score from the Perceptual Aberration and Magical Ideation scales above 3.0. There were 246 people in the control group (56.1% women, mean age 18.82 years, *SD* = 0.9, 89.4% Caucasian) who scored less than 0.5 *SD*s below the mean on the Revised SocAnh Scale, Perceptual Aberration Scale, and Magical Ideation Scale. There were no significance between group differences on sex or age, but the control group had significantly more Caucasian participants than the other two groups, *X*^2^ (2, *N* = 403) = 18.37, *p* < 0.001. Thus, we tested if there were ethnic group differences (Caucasian vs. non-Caucasian) on any of the variables of interest (implicit/explicit positive and negative affect, judgment) and found that there were none, all *p*s > 0.2. Given these findings, we did not consider ethnicity further.

### Measures

#### Explicit Affect

To assess current, explicit affect, participants were shown eight positively and eight negatively valenced words with both high (e.g., elated, anger) and low arousal levels (e.g., serene, sad). They were asked, “How are you feeling right now?” and were given a seven-point scale (0 = not at all to 6 = extremely strongly) to respond. These words have been used frequently in previous research to assess self-reported affect (e.g., Barrett, 2004). Cronbach’s α for the positive explicit affect measure was 0.78 and for the negative explicit affect measure was 0.86. One person in the PerMag group and one person in the Control group were omitted for missing responses to at least two of the mood items (due to either equipment failure or to having invalidly fast responses, reaction times < 200 ms; note that they were dropped without knowledge of their group membership).

#### Implicit Positive and Negative Affect Test (Quirin et al., 2009a)

To assess implicit positive and negative affect, participants completed the IPANAT. The IPANAT measures positive and negative affect in an indirect way by asking participants to rate the extent to which artificial words from a supposed artificial language conveys certain moods. Given their reliability over a period of a year, test–retest coefficients suggest a moderate to strong trait component of implicit positive and implicit negative affect as measured by the IPANAT. Also, as expected for implicit measures, correlations between implicit scores from the IPANAT and explicit state affect measures are generally in the low to moderate range (e.g., correlation between state Positive and Negative Affect Schedule scores and implicit positive affect, *r* = 0.20; Quirin et al., 2009a). Thus, because of their associations with trait and state measures, scores on the IPANAT reflect both trait and state variance. Lastly, relationships between scores on the IPANAT and physiological measures have consistently been in the expected direction and have accounted for effects above and beyond explicit measures of affect (e.g., decreased implicit positive affect and increased cortisol awakening response; increased implicit negative affect and higher systolic blood pressure; Quirin et al., 2009b; van der Ploeg et al., 2016).

In this task, participants saw six artificial words, and for each word, participants were asked to rate the extent to which the sound of the word conveyed each of the following moods: happy, helpless, energetic, tense, cheerful, inhibited on a four-point scale (1 = doesn’t fit at all to 4 = fits very well). Thus, there were 36 word pairs (each of the six words with each of the six mood words). Following Quirin and colleagues, scale scores were derived through a two-step process. First, scores for single mood adjectives were computed with the average of all six artificial word judgments that refer to the respective mood word (e.g., average ratings of “happy” when paired with each of the artificial words). Then, scores for implicit positive and implicit negative affect were computed by averaging adjective scores derived from positively valenced adjectives and negatively valenced adjectives, respectively. In the current study, Cronbach’s α for the implicit positive affect was 0.88 and for the implicit negative affect was 0.76. Three people in the Control group were omitted because they had at least four trials where their responses were invalidly fast, with reaction times <200 ms (note that they were dropped without knowledge of their group membership).

#### Judgment

Based on the judgment of risk likelihood task developed by Gasper and Clore (2000), which assesses perceived likelihood that negative events would occur in the near future, we created items to assess perceived likelihood that positive events would occur in the near future. On this task, participants were presented with five positive events (e.g., “How likely does it seem that you will achieve many of your future goals for the next 6 months?”) and were asked to rate the likelihood of each event happening to them compared to the average college student on a 10-point scale (0 = extremely unlikely to 9 = extremely likely). Please see the Supplementary Material for the full set of items. Cronbach’s α for the judgment measure was 0.61.

### Procedures

After informed consent was obtained, participants completed the IPANAT, the judgment task, and rating of current explicit affect, as well as personality measure not reported here. All measures were administered through E-Prime computer software (Psychology Software Tools Inc., 2006). This research project was approved by the Institutional Review Board at University of Missouri, and all procedures were consistent with the principles of ethical conduct of human research.

### Statistical Approach

For each affect measure, we conducted an omnibus repeated measures ANOVA. We followed up significant group effects with *t*-tests and calculated Cohen’s *d*s using group means and standard deviations as a measure of the magnitude of the differences between groups. A Cohen’s *d* value of 0.2 was considered a small effect size, 0.5 was considered a medium effect size, 0.8 was considered a large effect size, and a value >1.2 was considered a very large effect size (Cohen, 1988; Sawilowsky, 2009). When a significant two-way interaction was found, we created difference scores by subtracting scores on one subscale/measure from scores on the other subscale/measure (i.e., explicit positive affect minus explicit negative affect; implicit positive affect minus implicit negative affect) and then conducted *t*-tests in order to test the extent to which the relative differences between the measures within each group differed between the groups. Last, we tested for the relations of explicit and implicit positive affect and judgment, within each group and then examined whether the size of these association differed significantly between groups (Hays, 1988).

## Results

### Medium-to-Large Decreased Explicit Positive Affect in SocAnh and Medium-to-Large Increased Explicit Negative Affect in SocAnh and PerMag

**FIGURE 1:**
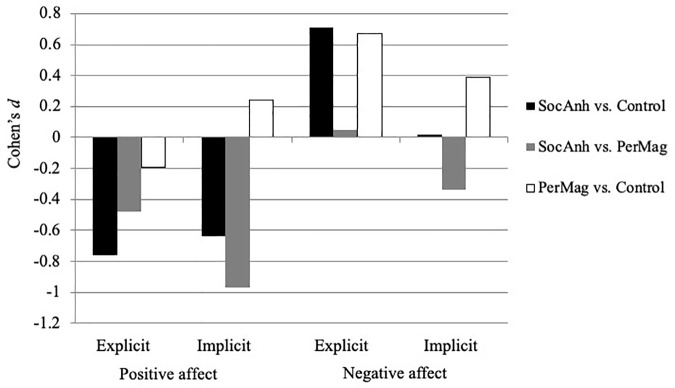
Effect sizes (Cohen’s *d*) for each group comparison on measures of explicit and implicit affect.

First, we examined levels of explicit positive and negative affect. As can be seen in Table 1 and Figure 1, there was a significant between-group difference on the explicit affect scores, *F*(2,398) = 3.9, *p* = 0.02, along with a significant group by affect scores interaction, *F*(2,398) = 35.49, *p* < 0.001. For explicit positive affect, the SocAnh group had significantly lower levels compared to both the Control group, *t*(338) = −6.24, *p* < 0.001, *d* = −0.76, and PerMag group, *t*(154) = −3.01, *p* = 0.003 *d* = −0.48, and these effects were medium-to-large in magnitude. For explicit negative affect, as expected, the SocAnh group had significantly higher levels compared to the Control group, *t*(338) = 6.33, *p* < 0.001, *d* = 0.71, and this effect was medium in magnitude. In addition, when comparing the explicit positive and negative affect scores, the SocAnh group’s explicit positive affect scores were relatively lower than their negative affect scores compared to the difference in the Control group, *t*(338) = −8.44, *p* < 0.001, *d* = −0.96, and this effect was large.

**Table 1:** Means (SDs) and group differences for questionnaire and task measures.

	SocAnh (*n* = 95)	PerMag (*n* = 62*)*	Controls (*n* = 246)
**Explicit affect**			
Explicit positive affect	16.27 (7.71)^a,b^	20.46 (9.55)^b^	22.18 (7.88)^a^
Explicit negative affect	18.05 (10.98)^a^	17.51 (10.61)^b^	11.23 (7.98)^a,b^
**Implicit positive and negative affect test**			
Implicit positive affect	1.94 (0.48)^a,b^	2.44 (0.55)^b^	2.30 (0.63)^a^
Implicit negative affect	1.93 (0.47)	2.08 (0.46)^a^	1.91 (0.40)^a^
Judgment	5.60 (1.27)^a,b^	5.89 (1.24)^b^	6.05 (1.19)^a^

*Matching superscripts indicate significant between-group differences.*

In contrast to SocAnh, the PerMag group did not differ significantly from the Control group in level of explicit positive affect, *t*(303) = −1.46, *p* = 0.15, *d* = −0.20, but similar to the SocAnh group, the PerMag group had higher explicit negative affect scores than the Control group, *t*(304) = 5.12, *p* < 0.001, *d* = 0.67, and this effect was medium in size. In addition, when comparing the explicit positive and negative affect scores, the PerMag group’s explicit positive affect scores were relatively lower than their negative affect scores compared to the difference in the Control group, *t*(304) = −4.48, *p* < 0.001, *d* = −0.58 (i.e., standardized score differences for PerMag vs. Controls = −0.38 vs. 0.51, respectively). At the same time, there were no significant relative differences between explicit positive affect and explicit negative affect scores for the PerMag and SocAnh groups, *t*(154) = −1.89, *p* = 0.06, *d* = −0.19, and this effect was small.

Overall, the SocAnh group reported decreased explicit positive affect, and both the SocAnh and PerMag groups reported increased explicit negative affect compared to the Control group. In addition, both the SocAnh and PerMag groups had relatively lower explicit positive affect scores vs. explicit negative affect scores compared to the Control group.

### Medium-to-Large Decreased Implicit Positive Affect in SocAnh and Small Increased Implicit Negative Affect for PerMag

Next, we examined levels of implicit positive and negative affect measured by the IPANAT. As can be seen in Table 1 and Figure 1, there was a significant between-group difference on the IPANAT affect scores, *F*(2,397) = 14.32, *p* < 0.001, along with a significant group by affect scores interaction, *F*(2,397) = 10.04, *p* < 0.001. For implicit positive affect, the SocAnh group showed lower levels than both the Control group, *t*(336) = −4.92, *p* < 0.001, *d* = −0.64, and the PerMag group, *t*(155) = −5.96, *p* < 0.001, *d* = −0.97, with these effect sizes medium-to-large magnitude. In contrast, for implicit negative affect, SocAnh did not significantly differ from the other groups and the magnitudes of such effects were small (vs. Controls, *t*(339) = 0.33, *p* = 0.74, *d* = 0.02; vs. PerMag, *t*(155) = −1.96, *p* = 0.052, *d* = −0.34). Moreover, comparing the implicit positive and negative affect scores, the SocAnh group’s level of implicit positive affect was lower than their level of implicit negative affect compared to the difference in both the Control group, *t*(336) = −4.53, *p* < 0.001, *d* = −0.58 (i.e., standardized score differences for SocAnh vs. Controls = −0.45 vs. 19, respectively), and the PerMag group, *t*(155) = −3.24, *p* < 0.01, *d* = −0.51 (i.e., standardized score differences for SocAnh vs. PerMag = −0.45 vs. −0.01, respectively). These effects were medium in size.

In contrast to SocAnh, the PerMag group did not differ significantly from the Control group in level of implicit positive affect, *t*(303) = 1.65, *p* = 0.10, *d* = 0.24, with if anything a trend for increased implicit positive affect in the PerMag group. Furthermore, as expected, the PerMag group reported significantly increased levels of implicit negative affect compared to the Control group, *t*(303) = 2.83, *p* = 0.01, *d* = 0.39, although the effect size was small in magnitude. Furthermore, comparing the implicit positive and negative affect scores, the PerMag group’s scores were not significantly different compared to the difference in the Control group, *t*(303) = −0.21, *p* = 0.84, *d* = −0.03.

Overall, the SocAnh group had significantly decreased levels of implicit positive affect, and these differences were medium-to-large in magnitude. In addition, the SocAnh group had relatively lower implicit positive affect scores vs. implicit negative affect compared to the Control group. At the same time, the PerMag group showed a small increased level of implicit negative affect compared to the Control group.

### SocAnh Gave Lower Positive Judgment Ratings Compared to Controls

Next, we examined whether the groups differed in judgment. As can been seen in Table 1, there was a main effect for group on judgment of likelihood of future positive events. The SocAnh groups had significantly lower likelihood ratings than the control group, *p* = 0.01, *d* = −0.37. There were no significant differences between judgment ratings of the SocAnh and PerMag groups, *p* = 0.34, *d* = −0.23, or between the PerMag and Control groups, *p* = 0.66, *d* = −0.13.

### Relations Between Judgment and Explicit Affect Differed by Group

First, we examined the relationships between judgments and current explicit positive and explicit negative affect within each group. As expected, for all groups, there was a significant relationship between judgment and current explicit positive affect, as people thought positive events were more likely when they were in a positive affective state (SocAnh: *r* = 0.42, *p* < 0.001; PerMag, *r* = 0.49, *p* < 0.001; Control: *r* = 0.21, *p* = 0.001^1^). Then, we tested whether the size of the association between judgment of likelihood of positive events and explicit positive affect differed significantly between groups. There was a significant difference between the size of the correlation, between the SocAnh and Control groups, *Z* = 1.92, *p* = 0.03, and between the PerMag and Control groups, *Z* = 2.13, *p* = 0.02. However, there was no difference in the size of the effect between the SocAnh and PerMag groups, *Z* = 0.45, *p* = 0.33. Overall, there were stronger associations between current explicit positive affect and judgment in the groups with elevated SocAnh or PerMag.

Similar to the results for explicit positive affect, there was a significant relationship between judgment and current explicit negative affect for all groups, as people thought positive events were less likely as their level of explicit negative affect increased (SocAnh: *r* = −0.21, *p* = 0.04; PerMag, *r* = −0.41, *p* = 0.001; Control: *r* = −0.22, *p* < 0.001). Then, we tested whether the size of the association between judgment of likelihood of positive events and explicit negative affect differed significantly between groups. Although there were no significant differences between the groups, there were trends for the PerMag group to show the strongest relationship (PerMag vs. Control groups, *Z* = 1.44, *p* = 0.07; PerMag vs. SocAnh groups, *Z* = 1.33, *p* = 0.09; Control vs. SocAnh *Z* = 0.11, *p* = 0.46).

### Stronger Relations Between Implicit Affect and Judgment in the PerMag Group

We then examined the relationships between judgments and current implicit positive and negative affect within each group. There was only a significant correlation between judgment and implicit positive affect in the PerMag group, *r* = 0.40 *p* = 0.001, but not in the SocAnh group, *r* = 0.11, *p* = 0.29, or Control group, *r* = 0.04, *p* = 0.54. Then, we examined whether the size of the association between judgment of likelihood of positive events and implicit positive affect differed significantly between groups. There was a significant difference in the size of the effect between PerMag and SocAnh groups, *Z* = 1.85, *p* = 0.03, and between the PerMag and Control groups, *Z* = 2.61, *p* = 0.005. However, the size of the effect did not differ between SocAnh and Control groups, *Z* = 0.58, *p* = 0.28. These results indicate that for the PerMag group only, their implicit positive affect was related to their judgment of likelihood of future positive events.

Similar to the results for implicit positive affect, there was only a significant correlation between judgment and implicit negative affect for the PerMag group, *r* = −0.33, *p* = 0.009, but not the SocAnh group, *r* = 0.12, *p* = 0.23, or Control group, *r* = −0.06, *p* = 0.34. Then, we examined whether the size of the association between judgment of likelihood of positive events and implicit negative affect differed significantly between groups. There was a significant difference in the size of the effect between PerMag and SocAnh groups, *Z* = −2.81, *p* = 0.003, and between the PerMag and Control groups, *Z* = −1.95, *p* = 0.03. However, the size of the effect did not differ between SocAnh and Control groups, *Z* = −1.51, *p* = 0.07. Again, similar to the results for implicit positive affect, these results indicate that for the PerMag group only, their implicit negative affect was related to their judgment of likelihood of future positive events.

## Discussion

Schizotypy has been previously associated with emotional dysfunction. However, the nature of this dysfunction is not well understood. The current study sought to clarify some of these abnormalities through an examination of different emotion facets (i.e., explicit and implicit positive and negative affect). The current study found that SocAnh was associated with both decreased explicit and implicit measures of positive affect. Moreover, the current study found that PerMag was associated with an increased influence of implicit affect on judgment. Hence, the current results suggest that an increased influence of implicit affect might foster the development of odd and unusual thoughts and experiences. Overall, consistent with prior research (e.g., Martin et al., 2012; Karcher et al., 2015), this current work suggests that there are unique emotional facet abnormalities between schizotypy dimensions.

The current study is also the first to show that deficits in positive affect associated with SocAnh are not limited to explicit measures of affect. Using a measure of implicit affect, we found that individuals with elevated levels of SocAnh showed medium-to-large decrements in implicit positive affect compared to the Control and PerMag groups. Thus, current results suggest that the finding of decreased positive affect in SocAnh is not limited to explicit measures of positive affect (Cohen et al., 2017; Li et al., 2019). Overall, the findings of the medium-to-large differences in both implicit and explicit affect in SocAnh suggest that a defining feature of SocAnh is decreased positive affect.

The findings of decreased implicit positive affect in SocAnh are consistent with some other findings suggesting SocAnh is associated with decreased responses in other positive affect measures. For instance, in an ERP study that examined the late positive potential (LPP), there was evidence of a decreased early response to positive stimuli in SocAnh, with SocAnh also associated with a relatively smaller LPP for positive than for negative stimuli (Martin et al., 2017, 2019). In another study that employed a linguistic analysis of a free writing period, SocAnh was associated with decreased used of positive words, even after a positive mood induction (Fung et al., 2017). Hence, the current study provides novel converging evidence that SocAnh is associated with lower levels of positive affect on a range of measures beyond explicit ratings of positive affect.

An important area for future research is to further examine the nature of decreased positive affect in SocAnh. For instance, it has been suggested that SocAnh might be related to decreases in the top-down or controlled processing of positive affective stimuli (Martin and Kerns, 2010; Martin et al., 2012, 2017). One way this could be examined would be to examine the neural correlates of processing positive stimuli in SocAnh and whether this is associated with decreased activity in brain areas associated with higher level conceptual appraisals of positive stimuli (e.g., lateral orbitofrontal cortex; Dixon et al., 2017). Another area of research might be to examine whether interventions that attempt to increase positive affect, such as teaching individuals to recall and savor positive emotional experiences (Johnson et al., 2011; Favrod et al., 2015), are effective in people with elevated levels of SocAnh and whether they could aid in the prevention of the development of schizophrenia-spectrum personality disorders.

In the current research, although SocAnh was associated with decreased positive affect across multiple measures, explicit positive affect was more strongly associated with judgments about the likelihood of future positive events in SocAnh than in controls. Hence, even though SocAnh was associated with decreased explicit positive affect, it appears that the SocAnh group was still less able to remove the influence of positive affect when making judgments. This appears consistent with previous research that has reported that SocAnh is associated with decreased attention to especially positive emotions (Martin et al., 2011). Furthermore, there is research that people with decreased attention to emotions can be especially unable to remove the influence of affect from judgment (Clore et al., 2001). Hence, the current study suggests that even though SocAnh is associated with decreased explicit positive affect, SocAnh is associated with an increased influence of explicit positive affect on judgment.

Similar to SocAnh, PerMag in the current study was associated with an increased association between explicit positive affect and judgment than was found in controls. However, the increased influence of affect in PerMag appeared to be much broader than in the other groups. Perhaps most importantly, in PerMag there were significantly stronger relationships between both implicit positive and implicit negative affect and judgment compared to the other groups, with the other groups not exhibiting an association between implicit affect and judgment. Hence, in PerMag, with increased implicit positive affect, people thought future positive outcomes were more likely to occur. Conversely, in PerMag, with increased implicit negative affect, people thought future positive outcomes were less likely to occur. The current results for PerMag appear consistent with previous evidence that PerMag is associated with people self-reporting being more influenced by their emotions (Cicero and Kerns, 2010). However, the current study for the first time has found evidence for this increased influence of affect on a judgment task. Moreover, the current study has found that this increased influence might be especially true for implicit measures, as only the PerMag group exhibited associations between implicit affect measures and judgment. Taken together, these findings are consistent with the idea that an increased influence of affect could be related to one’s “latent readiness for psychotic symptom formation” (LRPSF; Haralanov et al., 2015), a fleeting transition stage between the predisposition for developing psychosis and the schizophrenia genotype (i.e., *pathos et nosos schizophreniae*; Snezhnevsky, 1977). That is, the impact of affective information on how one judges and makes sense of the world could be causally related to one’s LRPSF.

Given that implicit affect is thought to reflect System 1 thinking that relies on heuristics and automatic spreading of activation (Quirin and Bode, 2014), this suggests that the increased influence of implicit affect on PerMag reflects an increased influence of System 1 thinking in PerMag. This seems very consistent with the theoretical view of Risen (2016) that odd beliefs and experiences in PerMag reflect an increased influence of System 1 thinking. One possible reason for this increased influence of implicit affect in PerMag is that implicit affect is increased in PerMag. Consistent with this, in the current study the PerMag group did have significantly increased implicit negative affect, with a trend for increased implicit positive affect, relative to controls. This is also consistent with other evidence that PerMag is associated with increased psychophysiological indices when processing both positive and negative affective stimuli (Karcher and Shean, 2012; Martin et al., 2017). However, it should also be noted that the increased implicit affect in the current study in PerMag involved small effect sizes. Another possibility, as has been theorized by Risen (2016), is that PerMag is also associated with decreased ability or an unwillingness to use more effortful and rational System 2 thinking to override System 1 thinking and the influence of implicit affect. Decreased ability or an unwillingness to engage System 2 thinking also seems consistent with evidence that PerMag is associated with decreased trait intellect (Chmielewski et al., 2014). Future research could investigate the extent to which implicit affect and intellect are related to other types of judgments and decision-making in PerMag, such as decisions in economic games, which has been found to be impaired in people with schizotypal traits (van’t Wout and Sanfey, 2011).

Overall, these results suggest that decreased positive affect is a core feature of SocAnh and that a heightened influence of affect could be related to the development of peculiar beliefs/experiences associated with PerMag. However, there are limitations of the current study. One involves the Cronbach’s α of the judgment task. Although the alpha value of the judgment measure in the current study is similar to one previously reported on a similar measure of judgment (Martin et al., 2011), it may appear relatively low compared to this study’s other included measures. Importantly though alpha is a function of the number of items on a scale. Scales with fewer items typically having lower alpha values than scales with more items, and thus, alpha it is not a direct reflection of the scale quality or its ability to measure the intended construct (Cortina, 1993). Thus, given its face validity and its associations with predicted outcomes (e.g., explicit positive affect), it appears to be an acceptable measure of judgment. Also, measures with lower reliability might attenuate the correlation between two variables (Nunnally, 1978), but this is unlikely to account for the differential size of the associations between the affect measures and judgments among the groups (i.e., this attenuation would affect the groups equally). Another limitation of the current study is the use of a college undergraduate sample, limiting the generalizability of the findings. However, previous longitudinal work (Chapman et al., 1994; Kwapil, 1998; Gooding et al., 2005) has found that college students with elevated levels of schizotypy are at greater risk for the development of schizophrenia-spectrum disorders and psychosis. Thus, this suggests the current findings do speak to abnormalities in schizotypy but need replication in community samples. Last, although neuroticism did not account for the relationship between positive mood and judgment, there are other variables that might influence one’s judgment of likelihood of positive events, such as depression and social anxiety (Muris and van der Heiden, 2006), that we did not measure. Future research could include such measure to test whether they account for the effects found in the current study. Despite these limitations, the current findings are novel and have potential prevention and intervention implications.

## Data Availability

The raw data supporting the conclusion of this manuscript will be made available by the authors, without undue reservation, to any qualified researcher.

## Ethics Statement

All study procedures were approved by the University’s Institutional Review Board. All participants provided written informed consent to participate.

## Author Contributions

EM conducted the statistical analyses and wrote the first draft of the manuscript. JH and KS were involved in the data collection. JK designed the study. All authors approved the final manuscript.

## Conflict of Interest Statement

The authors declare that the research was conducted in the absence of any commercial or financial relationships that could be construed as a potential conflict of interest.

## Footnotes

1 To test whether neuroticism (DeYoung et al., 2007) accounted for the relationship between positive mood and judgment, we ran partial correlations between positive mood and judgment controlling for neuroticism. Because we found the same pattern of results (SocAnh: *r* = 0.39, *p* < 0.001; PerMag, *r* = 0.43, *p* = 0.001; Control: *r* = 0.16, *p* = 0.012), we did not consider this further.
