# Supplementary material for: Explicit and Implicit Affect and Judgment in Schizotypy
Source: Front Psychol. 2019 Jul 1;10:1491. doi: 10.3389/fpsyg.2019.01491 (PMC6613436; doi:10.3389/fpsyg.2019.01491)
Supplement: Supplementary file 1 [file Table_1.DOCX]

**Supplementary Materials**

**Judgment task.**

Rate the likelihood of each of the following happening to you compared to the average college student on a 10-point scale (0 = extremely unlikely to 9 = extremely likely).

1. Compared to the average college student, how likely does it seem that you will achieve many of your future goals for the next 6 months?

2. Compared to the average college student, how likely does it seem that you will be satisfied with your professor or teacher's assistant because of a grade?

3. Compared to the average college student, how likely does it seem that you will say something that seems informed and intelligent to the people around you soon?

4. Compared to the average college student, how likely does it seem that you will have less than the usual difficulty getting your assignments and work done?

5. Compared to the average college student, how likely does it seem that you will do something that you are extremely proud of?
